# A community-based survey of visible congenital anomalies in rural Tamil Nadu

**DOI:** 10.4103/0970-0358.57191

**Published:** 2009-10

**Authors:** K. Sridhar

**Affiliations:** Past President APSI, Chennai, Tamil Nadu, India

**Keywords:** Cleft lip, Cleft palate, Community survey, Epidemiology of facial cleft, Visible birth defects

## Abstract

An extensive community-based survey of visible congenital defects covering 12.8 million children in rural Tamil Nadu state was conducted during the years 2004–05. A door-to-door survey was done utilizing the existing health care delivery system. More than 10,000 village health nurses were involved to collect the data. All children between the ages of 0 and 15 years were seen.

The children with defects were seen by a medical officer and diagnosis was made as per chart. A total of 1.30% of children were born with some visible anomalies. The male:female ratio was 1.3:1. There was a family history in 9% and consanguinity in 32%. More than 5% mothers had taken some medication in the first trimester of pregnancy out of which anti-convulsants were 3.4%. Facial clefts showed a lower incidence of 1 in 1976 live births with peak incidence between March and June. Cleft palate alone showed a higher percentage (30%) than other studies.

## INTRODUCTION

Sayetta, Weinrich and Coston[1] described the problems associated with epidemiological studies in orofacial clefts: ‘Case findings using data sources such as birth certificates, foetal death certificates, and hospital records often produce ascertainment bias, selection bias, or both’. A variety of reasons are advanced to explain the wide discrepancies in reported statistics on orofacial clefting from different geographic areas, ethnic groups and time periods.

Most of the studies are based on hospital or birth registry statistics which do not reflect the true incidence of the disease. Even though community-based surveys may reflect close to true incidence, they are difficult to conduct. It requires a large knowledgeable work force, is time consuming and expensive.

Proper statistics on birth anomalies in India were not available and we decided to do a community-based survey. The Association of Plastic Surgeons of India appealed to the government of Tamil Nadu to help in the community-based survey utilizing the structure of the health care delivery system of the state. To make the best use of the work force we decided to do the survey to encompass all visible birth defects in rural Tamil Nadu among children between the ages of 0 and 15 years. After ascertaining the number of children affected, the government wanted to put in place a system to take care of the treatment for these children. Initially it was decided to take up treatment for cleft lip and palate at teaching hospitals with available plastic surgeons.

## MATERIALS AND METHODS

We chose rural areas instead of urban as urban areas have a large floating population and are not covered under any single specific health system. We used the existing health workers of Tamil Nadu, under the Director of Public Health and Preventive Medicine.

The total population of Tamil Nadu during the period of survey was 65.5 million. Out of this, the rural population covered by the system was 47.2 million. Children up to 15 years of age amounted to 12.8 million. Our survey was confined to this population.

### The basic infrastructure of health care in rural Tamil Nadu

The structure of health care delivery system in rural Tamil Nadu is one of the best in the country [Table 1]. Tamil Nadu's preventive health system is under a Director of Public Health and Preventive Medicine with an Additional Director and a Joint Director of primary health centre (PHC) to help him. The state has a Bureau of Health Intelligence under the public health service headed by a Joint Director of Statistics.

**Table 1:**
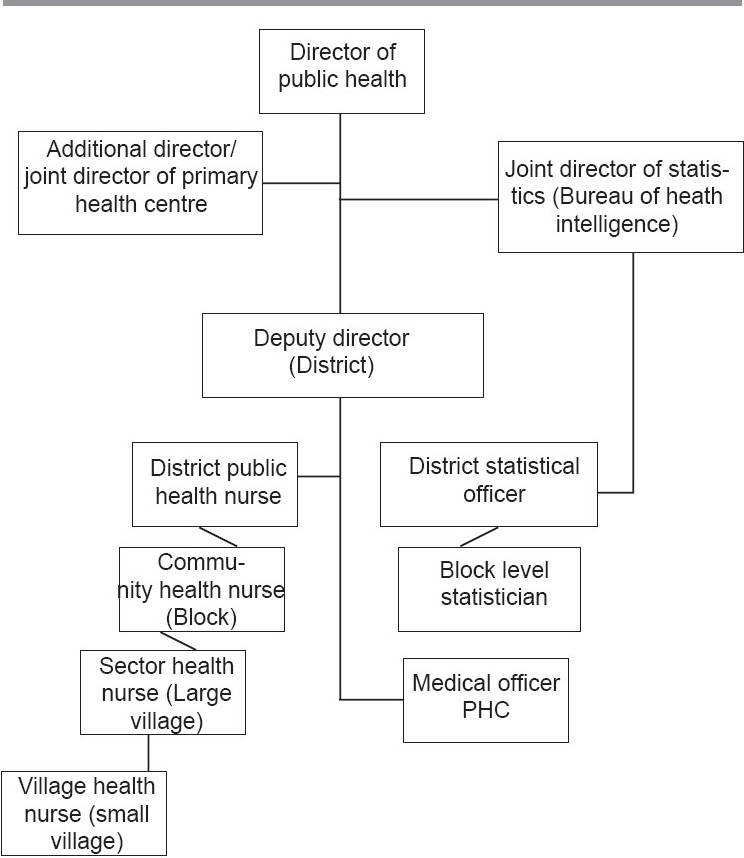
Staff of Director of Health Services, Tamil Nadu, India

#### District

**Figure 1:**
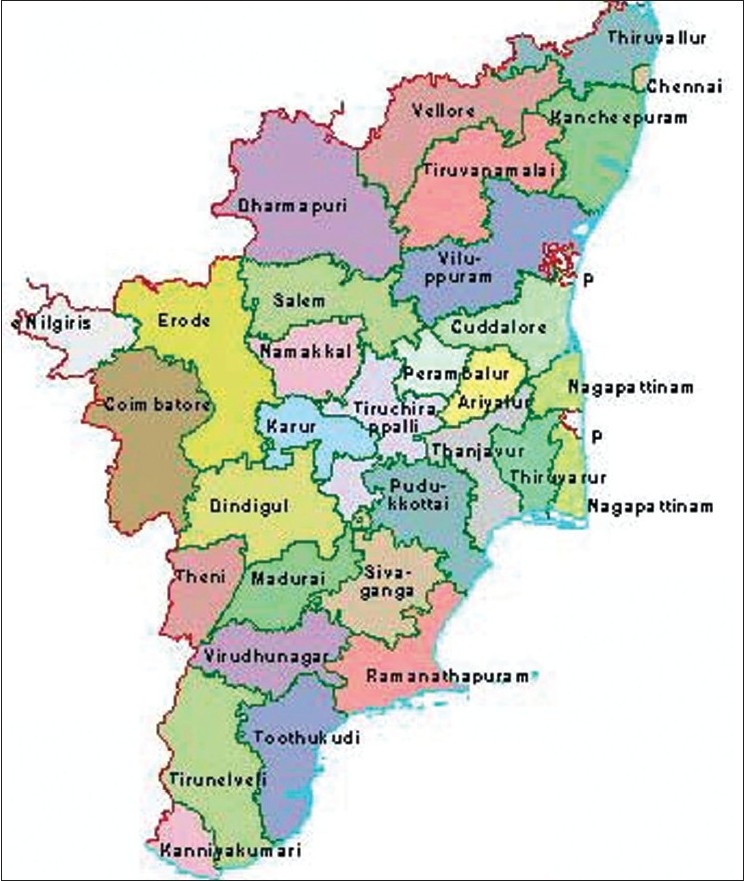
Districts of Tamil Nadu, India

There are 30 revenue districts in Tamil Nadu [Figure 1]. These 30 revenue districts are further divided into 42 health unit districts (HUD) of about 1.5–2 million populations. Each district is under the control of Deputy Director of Health Services. It has a District Public Health Nurse (DPHN) and a district-level Statistical Officer.

#### Block

Every district comprises around 15–16 blocks. Each block covers a population of about 100,000. It has a Community Health Nurse (CHN) and a block-level Statistician. There were in total 288 CHNs during the period of survey.

#### Primary Health Centre

Every block comprises about three to four primary health centres located in larger villages of 30,000 or more population. It is served by two medical officers and Sector Health Nurse (SHN). 2378 medical officers and 1175 SHNs took part in the survey

#### Village sub-centre

Smaller villages under the primary health centres are covered by village sub-centres which cater to a population of about 5000 or little more. Each sub-centre is managed by a village health nurse (VHN). She lives in the same community and knows each and every family. Every VHN covers about 1000–2000 families. There were 10,196 VHNs who took part in this study.

#### Survey

Every VHN maintains a family register containing particulars of every family member under her area. She visits the families regularly and collects information and implements various welfare schemes of the government eg. family welfare, folic acid and iron supplementation for women etc. Every Tuesday, all the VHNs under the PHC meet at the respective centres and report to the medical officer and the Sector Health Nurse. We utilized this system for our study. The VHNs were asked to cover in person all the families under them. They were asked to see all the children up to 15 years of age in their respective area.

#### Pre-survey training

We trained all the VHNs involved in the survey at the block level. They were all given an illustrated hand book.

#### Questionnaire and physical examination

Every child, whether normal or not, had a form to be filled up [Table 2]. The first part of the form contained general details about the child like age, sex, child's unique number and so on. The next part contained questions regarding symptoms associated with various defects like speech, urination, fits. The third part contained a list of various possible sites of birth defects like head, lip, palate, genitalia, hand and others. If the child had any defect, then the nurse proceeded to fill the latter part of the form which contained details of antenatal, perinatal, and family history. Specific questions regarding factors known to have effect on birth defects were included in this form. The forms were in Tamil which is the local language, and the English version is given in Table 1. The village nurse just ticked off the site of involvement. For example, if the child had cleft lip, the nurse would just tick off opposite ‘lip’; but if the child had hypospadias, she would put a tick against ‘genitals’. She did not make any attempt to diagnose. It was made clear to the VHN that details for operated and not operated children need to be recorded. Details of any treatment especially surgeries done were recorded including the place of surgery [Table 2].

**Table 2:** Form used by a VHN to fill-up personal details of children, history and details of defect

FORM -I Number											

*District No.*		*Block*		*Pri. Health No.*		*Sub Center (PHC)No.*		*Village No. (HSC) No.*		*Child No.*	
09	1	1	0	0	1	0	2	0	4	10	03
(The boxes above are to be filled with numbers assigned for the district/block/PHC/HSC /village/ and the child number is the number for the family + the number denoting the child in family register e.g. If the revenue district is. 09/health unit district 1/block10/PHC 01/HSC02/Village04/Family10 + number assigned to child in family is 03 then the number is 091100102041003. This becomes the unique number for the child).											
**BIRTH DEFECT SURVEY**											
Primary health center name………………					Subcenter name………………………..						
Village name……………………..					Name of village Health Nurse…………						
					(Who collected the data)					
**1. Details of affected Child**											
Number designated for this child in family record………											
(In Family records if father is 01, mother 02, first child 03, second child 04,											
grand mother 05 and this affected child is the first child, then give the number as 03)											
Name of child……………………….Age…………Sex……………Date of Birth………											
Mothers Name……………………….Fathers Name……………………………											
**2. Does the Child Has any of the following? If yes** 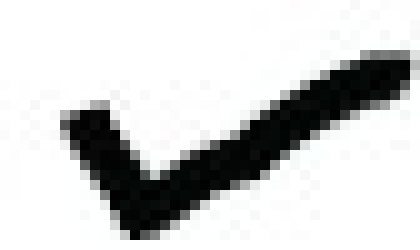 **mark**											
- Problems with speech											
- Convulsions											
- Defects in movements of hand or legs											
- Incontinence of urine / Motion											
- Breathlessness											
- Cyanosis											
- Mental Retardation											
**3. Anatomical area of defect**											
Head											
Eye											
Eye lid											
Nose											
Cleft Lip											
Cleft Palate											
Both Cleft Lip and Palate											
Any other problems in Lip											
Any other in Palate											
Tongue											
Ear											
Neck											
Chest											
Hand (Upper Limb)											
Leg (Lower Limb)											
Genitalia
Anus
Spine
Jaw
Any other area not mentioned
specify …………………
**Dates of surgeries**	**Name of the hospital**	**Part operated**									
									
4. Did the child undergo any surgery? If so, give details											
5. Any other relatives in the family who have similar defects (e.g. uncle, aunt or their children)	
a.	b.	c.						
6. Details of the prenatal and antenatal history											
Age of mother during delivery………											
Delivery at	(a) full term…………	(b) premature ………weeks								
Did the mother take any medication for fits? Yes/No											
Did she take iron folic acid as per scheme? Yes/No											
Did she take any other medication during the antenatal period? Yes/No											
If yes, for what? ……………………											
When?	Trimester	I ……. II……. III……….								
Was any X-ray taken during pregnancy? Yes/No											
If yes, when?	Trimester I……II………III										
Did the mother have any fever during pregnancy? Yes/No											
If yes, when?	Trimester I………..II………….III										
Was the delivery normal? Yes/No											
If no, give details…………………………….. (e.g. forceps/vacuum/Caesarean)											
6.09 village health nurse to furnish data of the village covered by her											

After completing the survey, she also filled up Form IA which contained agewise details about the village under her care eg. the population, sex ratio etc. [Table 3]. The number of children who died during the past 15 years and the still births in her area of survey as per family health records were mentioned in Form IA.

**Table 3:** Birth Defect Survey - Form 1A

**Form IA**												
**BIRTH DEFECT SURVEY**												
(Fill up 1–4 from Sub-centre Register and 5–6 from Survey Form-I)												
Village Health Nurse's Consolidated Form												
District……………. Block …………….. PHC……………. HSC…………….												
Village												
**1. Population of this village**												

***Up to 1 year***		***1–4 years***		***5–15 years***		***16–49 years***		***Above 50 years***		***Total***		

Male	Female	Male	Female	Male	Female	Male	Female	Male	Female	Male	Female	Total
2. Total number of families……………………………												
Number of families with defective children………….												
Number of families without defect……………………												
3. Religion: Hindu……… Muslim……….. Christian……….Others………….												
4. Scheduled caste: Male………..Female………….Total…………….												
5. Details of children below 15 years of age												
Defective: Male……….Female …………Total………………												
Normal: Male……….Female………… Total………………												
Total: Male……….Female…………												
If any child has died in the family within 15 years, give details												
Age at death………… Cause of death…………..												
Did the child have any defect? Specify												

During her weekly meetings at the PHC with the medical officer, she periodically brought the affected children along as and when she completed the survey in a particular area. The medical officer examined the children and completed the form. This form contained details of diagnosis like cleft lip, palate or both or clubfoot.

The forms were sent to the block level and thereafter to district headquarter through the statisticians. The district statistical officer compiled the data and despatched it to the directorate.

Cross-verification of random areas was conducted. Areas from which the distribution of anomalies showed a marked difference were double checked.

Sample analysis of part of one district was done. Apart from this, all the cleft lip and palate defects were earmarked and those who had not yet been operated at district hospitals and private hospitals under the Smile Train Programme.

## RESULTS

The total population covered was 47.2 million [Table 4]. Out of this, the number of children with the ages of 0–15 years were 12,818,691 (about 27%) including those who had died in the corresponding period. The village health nurses noticed 166,833 children with birth anomalies among these 12.81 million children, and brought them to the medical officer for further evaluation. This amounts to 1.30% of all live births having visible congenital anomalies. Among the children affected with anomalies 57.4% were males and 42.59% were females. The male:female ratio was 1.3:1. On the other hand, among the general population in Tamil Nadu, males form only 49.80% and females form 50.20%.

**Table 4:** Population and delivery statistics

Population	47.2 million
Children 0–15 years of age	12,818,691
Male	49.80%
Female	50.20%
Children with birth anomalies	166,833
Male children with anomalies	57.4%
Female children with anomalies	42.59%
Normal deliveries	96.9%
Vacuum/Caesarean section	3.1%
Full term deliveries	3%
Premature	3%
Institutional deliveries	82.27%
Domestic deliveries	17.73%

Family history was present in 9.05% patients.

The history of consanguinity could be elicited in 32% of children affected. In rural areas, the prevalence of consanguinity in the normal population was similar.

It was interesting to note that 1.15% attempted abortions in their first trimester. The ingestion of anti-epileptic medication was noted in 3.4% and analgesics in 1.5%.

A total of 5.3% of mothers had fever in the first trimester for which all took some medication or the other, probably analgesics and antipyretics.

All women received iron folic acid tablets in rural Tamil Nadu under the welfare scheme and surprisingly the compliance was 96%.

### Details of delivery

During this survey we also found out about the nature of delivery.

Whereas 97% pregnancies completed full-term, 3% delivered a premature child. 96.9% were normal deliveries, while 3.1% underwent vacuum assisted or Caesarean delivery. 82.27% were institutional deliveries while 17.73% delivered at home.

One district showed a high incidence of congenital anomalies compared to others alongwith an extremely high recorded death rate in the neonatal period. This raised doubts about infanticide in children with anomalies.

For this paper, three geographical areas wherein a cross-verification of the authenticity of data could not be done were totally eliminated from analysis. From a total of 167,750 visible anomalies, 11,181 from these areas were removed from analysis amounting to 156,569 after correction. The corresponding child population of 946,822 from 12.81 million was also left out. This study comprised of a total of 11.87 million children.

Of the visible anomalies after verification 2334 children had cleft lip, 1819 had cleft palate and 1855 had both cleft lip and palate with a total of 6008 children having these anomalies. Cleft lip and palate comprised 3.83% of all visible anomalies. 48 children with these anomalies died during the period of survey. 68 children with these anomalies had migrated elsewhere and could not be followed up for further treatment. Isolated cleft lips were noticed in 1 in 5086 births.

1 in 6526 live births had clefts of the palate and combined clefts of lip and palate were noted in 1 in 6399 live births.

If we leave out pure cleft palate patients, then the incidence seems to be 1 in 2834. This is the incidence of cleft lip with or without palate. Put together, both cleft lip and palate have an incidence of 1 in 1976 live births. Left-sided clefts were 10 times more common in unilateral clefts than right.

The ratio between unilateral and bilateral clefts was 10:3.

Out of these children, only 3794 children had undergone surgery prior to the survey. The remaining were motivated to undergo surgery. By 1 year, 5651 underwent surgery for both lip and palate.

We studied the period of child birth in two sample areas of districts to ascertain the seasonal variation in incidence, if any. The months of January and February showed a low incidence whereas the period March to June showed elicited a higher incidence for both cleft of the lip and palate.

## DISCUSSION

Jensen et al.[2] reported the incidence of cleft lip and palate in Denmark during a 5-year period between 1976 and 1981. According to them, the incidence of facial cleft was 1.89 per 1000 live births or 1 in every 529 live births. The sex distribution was 61% males and 39% females. Of these, 34% had only cleft lip, 39% had cleft of the lip and palate and 27% had only cleft of the palate.

Murray et al.[3] in their study in Philippines also report the incidence of 1.94 per 1000 which is equal to about 1 in every 515 live births.

Our studies show a comparatively lower incidence of facial clefts of 1 in 1976 live births. Only the Nigerian study of Iregbulem[4] reported a lower incidence of 1 in 2703 births.

Both the Philippine and Nigerian studies were based on perusing hospital records of consecutive births in one hospital.

The sex distribution in our studies also shows a preponderance of afflicted males compared to females. The ratio of males to females was 1.3:1 compared to 1.5:1 in the Denmark study.[2]

We also noted that the ratio of overall congenital malformations remained the same. It is interesting to note that among the general population in Tamil Nadu state, males were marginally less than females and formed 49.8% of the population.

Our study showed a higher incidence of cleft palate alone (30.27%) compared to the other studies in which it varied from 19% to 27%. The Chang Gung Memorial Hospital study[7] also showed an incidence of 30.88% for cleft palate without associated cleft of the lip like ours. Cleft lip alone formed 38.84% and cleft of the lip together with the palate formed 30.87%.

Attempted but failed abortion in rural area showed a high incidence of anomalies. This finding corroborates well with the findings of Vanderas[5] and Saxén.[6] Another notable feature is the consumption of anti-epileptic drugs (3.4%) in the first trimester.

We found the months of January and February showing a low incidence and March to June showing a higher incidence for both cleft of the lip and the palate. The study by Coupland[8] showed a higher incidence of cleft palate during August and September but cleft lip with or without palate showed a peak incidence between December and January. The Chang Gung Memorial Hospital study[7] also showed a similar higher incidence like Coupland.[8]

## CONCLUSION

This study was one of its kind in our country and we could not find a similar study elsewhere in the literature.

Our study showed a much lower incidence of facial clefts compared to other studies. Being a community-based study, our report mirrors the true incidence of cleft lip and palate in our population. Since the study involved massive collection of data, the number of personnel involved in data collection was also huge. Even if we give a margin for human error in collection and interpretation, the findings suggest a low incidence in our state. The compliance of taking of folic acid tablets by women of reproductive age as per records is good. Whether this has any role to play is worth studying.
